# Complete Genome Sequence of Pseudomonas aeruginosa CMC-115, a Clinical Strain from an Acute Ventilator-Associated Pneumonia Patient

**DOI:** 10.1128/MRA.00595-20

**Published:** 2020-07-23

**Authors:** Adenike Adenikinju, Roderick V. Jensen, Thomas M. Kerkering, Dorothy C. Garner, Jayasimha Rao

**Affiliations:** a Division of Infectious Disease, Virginia Tech Carilion School of Medicine, Roanoke, Virginia, USA; b Department of Biological Sciences, Virginia Tech, Blacksburg, Virginia, USA; c Internal Medicine, Division of Infectious Disease, Carilion Medical Center, Roanoke, Virginia, USA; Loyola University Chicago

## Abstract

We report the complete genome of clinical strain Pseudomonas aeruginosa CMC-115, which was isolated from an acute ventilator-associated pneumonia patient. Illumina sequencing reads were assembled using Geneious to yield a 6,375,262-bp circular chromosome that exhibited an unusual ferrichrome receptor in the pyoverdine synthesis locus and the absence of type 3 secretion system genes.

## ANNOUNCEMENT

Pseudomonas aeruginosa is an increasingly common opportunistic pathogen causing infection in immunocompromised and hospitalized patients, leading to health care-associated pneumonia. Patients on ventilators are particularly vulnerable; *P. aeruginosa* invades the lower respiratory tract and leads to ventilator-associated pneumonia (VAP), increasing the risk of death by as much as 30% in intensive care units (1–3). The complete genome of a *Pseudomonas* strain from an acute VAP case provides useful information for identifying novel virulence factors and antimicrobial resistance (AMR) genes.

A prospective study was conducted in 2010 to 2012 with approval from the Carilion Clinic Institutional Review Board. In that study, CMC-115 was obtained from the Quest Diagnostics Microbiology Laboratory at Carilion Clinic. The strain was isolated from a tracheal aspirate sample from an acute VAP patient, and its identity was confirmed by biochemical tests. Antimicrobial susceptibility testing was performed using a Vitek 2 automated system (bioMérieux) according to Clinical and Laboratory Standards Institute guidelines (4). The isolate was grown on a blood agar plate and transported to the Carilion Basic Science Laboratory (CBSL); glycerol stocks were prepared and stored in a −80°C freezer. The patient was treated at Carilion Roanoke Memorial Hospital and survived.

A single colony of CMC-115 was grown for 18 h in 25 ml lysogeny broth at 37°C, at 200 rpm. The cell pellet was used for genomic DNA isolation with a Genomic-tip 20/G (Qiagen) (5). The genome was sequenced on the Illumina NextSeq platform at the Virginia Tech Genomics Resource Center, with a library constructed using the Illumina TruSeq DNA preparation kit. Sequencing generated 68,209,004 paired-end reads with a length of 150 bp, which were assembled with the Geneious v11.0.4 (Biomatters, Ltd.) *de novo* algorithm to produce 40 contigs with a length of >1,000 bp and an *N*_50_ value of 423,776 bp. The Geneious map to reference algorithm, with fine tuning set to iterate up to 10 times, was then used to iteratively map reads to the ends of the 40 contigs until the contigs overlapped and closed into a circular genome. A final use of the Geneious map to reference algorithm aligned >95% of the reads to the complete circular genome, with a uniform average coverage of 1,500×. All Geneious assemblies and alignments were performed with the default low-sensitivity/fastest settings, which allow at most 10% base mismatches.

The complete assembly of CMC-115 resulted in a circular genome of 6,375,262 bp with a G+C content of 66.4%. The NCBI Prokaryotic Genome Annotation Pipeline v4.2 (6, 7) identified 5,930 genes, including 5,850 protein-coding genes and 80 RNA genes (64 tRNAs, 12 rRNAs, and 4 noncoding RNAs), along with 68 pseudogenes and 2 CRISPR arrays.

Interestingly, the type 3 secretion system and its effector genes *exoS*, *exoT*, *exoU*, and *exoY* are absent in the genome of CMC-115 (8, 9). The genome of CMC-115 contains many insertion sequence (IS) elements (10), representing the IS*3*, IS*4*, IS*21*, IS*63*, and ISNCY families (11, 12) (Table 1). We found an ∼101-kb locus for pyoverdine synthesis that contains an unusual *tonB*-dependent siderophore (ferrichrome) receptor (GO602_12470), the *fpvAIIb* (13–15) linked to IS*3* (GO602_12475). Chromosomal AMR genes were identified using the RGI v5.1.0 and CARD v3.0.8 (16) Web servers with default parameters. Ten genes were found, including two β-lactamase genes (*bla*_OXA-50_ and/or *bla*_OXA-395_ and *bla*_PDC-167_ and/or *bla*_PAO_), two phenicol resistance genes (*catB2* and *catB7*), an aminoglycoside resistance gene [*aph*(*3′)IIb*], a fosfomycin resistance gene (*fosA*), a vancomycin resistance gene (*vanWG*), a fusaric acid resistance gene (*fusc*) (17), a sulfonamide resistance gene (*sul1*), and a bleomycin resistance gene (*acetyltransf_10*) (18). In addition, 33 resistance-nodulation-cell division (RND) genes that are involved in antibiotic efflux mechanisms (19) and 5 genes involved in antibiotic target alterations were identified.

**TABLE 1:** Transposase family sequences in Pseudomonas aeruginosa CMC-115

Transposase family	Gene location	Locus tag	IS^*a*^	Origin^*b*^	Accession no.
IS*3* (ISPa57)	296203..297536	GO602_01345	IRL: TGTCTAGTCCTGAAATAGGTTTACACCTGCTCAGGTAGGCCGATAGGCCTCAAAACGCCCGAT; IRR: TGTCCCGCGCCAGATGGAGTTTTCGTCAGATGACAAGGACAGGTGACCTACAAGC	*P. aeruginosa* DK1, *P. aeruginosa* PA7	LN870292
IS*3* (ISPa57)	410967..412186	GO602_01860	IRL: TGTCCCGTCCTGAATAAGGTTTACACCTCCCATGCCGATTTTCCGGAGAGCTAGATGGAGCAA; IRR: TGTCTAGTCCTGAAATAGGTTTACACCTGCTCAGGTAGGCCGATAGGCCTCAAAA	*P. aeruginosa* DK1, *P. aeruginosa* PA7	LN870292
IS*3* (ISPa57)	2029342..2030561	GO602_09640	IRL: TGTCTAGTCCTGAAATAGGTTTACACCTGCTCAGGTAGGCCGATAGGCCTCAAAACGCCCGAT; IRR: TGTCCCGTCCTGAATAAGGTTTACACCTCCCATGCCGATTTTCCGGAGAGCTAGA	*P. aeruginosa* DK1, *P. aeruginosa* PA7	LN870292
IS*3* (ISPa57)	2674283..2675502	GO602_12475	IRL: TGTCTAGTCCTGAAATAGGTTTACACCTGCTCAGGTAGGCCGATAGGCCTCAAAACGCCCGAT; IRR: TGTCCCGTCCTGAATAAGGTTTACACCTCCCATGCCGATTTTCCGGAGAGCTAGA	*P. aeruginosa* DK1, *P. aeruginosa* PA7	LN870292
IS*3* (ISPa57)	4870826..4872045	GO602_22695	IRL: TGTCTAGTCCTGAAATAGGTTTACACCTGCTCAGGTAGGCCGATAGGCCTCAAAACGCCCGAT; IRR: TGTCCCGTCCTGAATAAGGTTTACACCTCCCATGCCGATTTTCCGGAGAGCTAGA	*P. aeruginosa* DK1, *P. aeruginosa* PA7	LN870292
IS*3* (ISPa57)	4953930..4955149	GO602_23110	IRL: TGTCTAGTCCTGAAATAGGTTTACACCTGCTCAGGTAGGCCGATAGGCCTCAAAACGCCCGAT; IRR: TGTCCCGTCCTGAATAAGGTTTACACCTCCCATGCCGATTTTCCGGAGAGCTAGA	*P. aeruginosa* DK1, *P. aeruginosa* PA7	LN870292
IS*21* (ISDev1)	858147..859679	GO602_04065	IRL: TGTTGGTTTGCACGCGGAACTGAGCCAGTTTTTCCACCGAGAAGTGAGCCACCTCTA; IRR: TGTTGGTTTCCACGCAGAACTGAGCCAATTAGGCAGATAATTTCCATTGA	*Devosia* sp. I507, *Aquamicrobium* sp.	CP026747
IS*3* (ISPa86)	11568120..1157562	GO602_05530	IRL: TCTACCCCGTTACCACGGACATTTTCGAGTAAGCTCACGCTGAGCTGAAGGAGTA; IRR: TGCGCCAACGCCTGGCTCGGTTGTCGCGCGCGGTAGCCACCTCGCTGCG	*P. aeruginosa*	MF344569
ISNCY (ISPa6)	1327230..1327763	GO602_06370	IRL: TGGTGTTGGTGTCGTGCCACGAGGGGATGAATAGGGTGGGTTGAAAAACGAATG; IRR: TGTGCGGGGTTCTTCATTAAAGAGAAATAAAAACCCAAGGTGTTCA	*P. aeruginosa* PAO-muc	U16784
IS*4* (ISPen1)	2789284..2790672	GO602_12805	IRL: TGGCACTTACTTAACCCTTTTCGGATGACCGTGTTTCTTGATCGCTGCCCGAGCAGATT; IRR: TGGCACTTACTTAAGCGCTAAGCGATTGATTCAGAAAAGAAAGGCTG	*Pseudomonas entomophila* L48	NC_008027
IS*3* (ISPa32)	3154955..3156092	GO602_14355	IRL: TGACCTGCTCCCCGATTTCAGTACCACGCCGAACTTAGTAGAGTCCGTTTTCCGAGCAG; IRR: TGACCTGCCCCCAGTTTTAGTACCGCTGCCTAGTTAGTAGGTCCTGGGGT	*P. aeruginosa* PAO1	NC_002516
IS*3* (ISAeca4)	5918861..5919667	GO602_27515	IRL: TGATGCTTAGCTGCCTTCCTCGTCTCCGATAAAGGTCCGGCCCAGCGCCGCTTGAGAAAC; IRR: TGAAATCGGGGGGCAGGTCACCGGCTGCTGCGGGTATCCCGCAGCGGCT	*Aeromonas caviae*	CP024198^*c*^
IS*3* (ISPa32)	5919740..5920878	GO602_27520	IRL: TGACCTGCCCCCCGATTTCAGTACCACGTCAAACTTAGTAGAGTCCGTTTTCCGAGCAG; IRR: TGACCTGCCCCCAGTTTTAGTACCGCTGCCTAGTTAGTAGGCCCTGGGGT	*P. aeruginosa* PAO1	NC_002516

a IRL, left inverted repeat; IRR, right inverted repeat.

b ISfinder tool (http://www-is.biotoul.fr) with the standard parameters (10).

c Accession number from an NCBI protein BLAST search.

### Data availability.

The annotated complete genome assembly of strain Pseudomonas aeruginosa CMC-115 is available in GenBank under accession numbers CP046602, SRR11592953, PRJNA593818, and SAMN13451386.
